# Fibrous Hamartoma of Infancy: A Case Report

**DOI:** 10.7759/cureus.103691

**Published:** 2026-02-16

**Authors:** Larbi Benradi, Mohamed Belahcen

**Affiliations:** 1 Pediatric Surgery, Mohammed VI University Hospital of Oujda, Oujda, MAR; 2 Pediatric Surgery, Faculty of Medicine, Mohammed VI University Hospital of Oujda, Mohamed I University, Oujda, MAR

**Keywords:** fibrous hamartoma, infant, soft tissue tumor, surgery, wrist

## Abstract

Fibrous hamartoma of infancy (FHI) is a rare, benign soft tissue tumor usually seen in the pediatric population under two years of age. The most usual localizations are the axilla, extremities, urogenital area, and abdomen. CT and MRI play an important role in determining the origins and analyzing the surrounding structures. The first-line treatment in FHI is local excision. A one-year-old male infant presented with swelling of the right wrist. On examination, the mass was solid, mobile, measuring 5 × 4 cm, and located in the soft tissue of the right wrist. Ultrasound was performed and showed a heterogeneous, poorly circumscribed swelling. MRI demonstrated no malignant features. A biopsy was performed, and pathological examination with immunochemistry showed FHI. The lesion was treated by marginal excision, and no complications were noted postoperatively. FHI presents as a slow-growing soft tissue swelling seen under the age of two years. MRI is the best examination to guide the diagnosis. Local excision is the most effective treatment.

## Introduction

Fibrous hamartoma of infancy (FHI) is a rare, slow-growing benign tumor arising from the soft tissue, usually seen under the age of two years; however, it can also occasionally present at birth [1]. The most common localizations are the abdomen, urogenital area, axilla, and shoulder. Males are more affected, especially during the first two years of life [2]. The clinical presentation often demonstrates a well-limited and pain-free soft tissue swelling. CT and MRI are useful in determining histological origins and ruling out other differential diagnoses. Curative treatment is surgical excision, with local recurrence being rare [3]. Here, we report a case of FHI in a one-year-old infant arising from the right wrist, posing a diagnostic challenge given the rarity of both the entity and its anatomical location. The patient was treated at our primary academic center. Clinical and radiological follow-up at 12 months showed no evidence of recurrence.

## Case presentation

**Figure 1:**
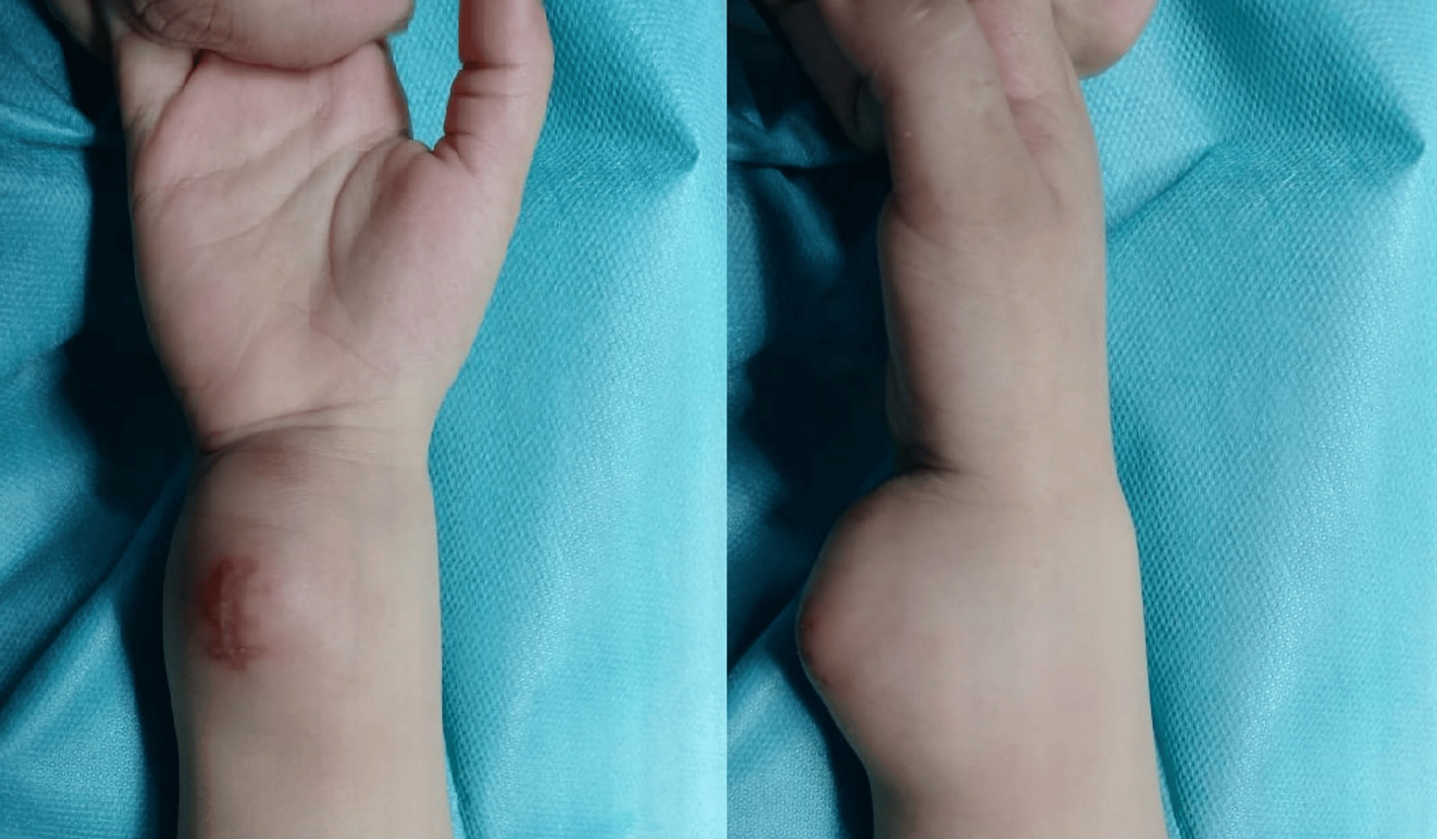
A solid, well-circumscribed, superficial mass on the anterior aspect of the right wrist, measuring approximately 5 × 4 cm.

A one-year-old child born following an unmonitored pregnancy and vaginal delivery with cephalic presentation was admitted to our department for a right wrist swelling noted six months ago, which had gradually increased in volume. Clinical examination revealed an infant in good general condition with a palpable mass located on the anterior surface of the right wrist, measuring 5 × 4 cm. The swelling was solid, painless, mobile, and well-limited (Figure 1). The rest of the clinical examination did not reveal any abnormalities, including the examination of lymph node regions.

**Figure 2:**
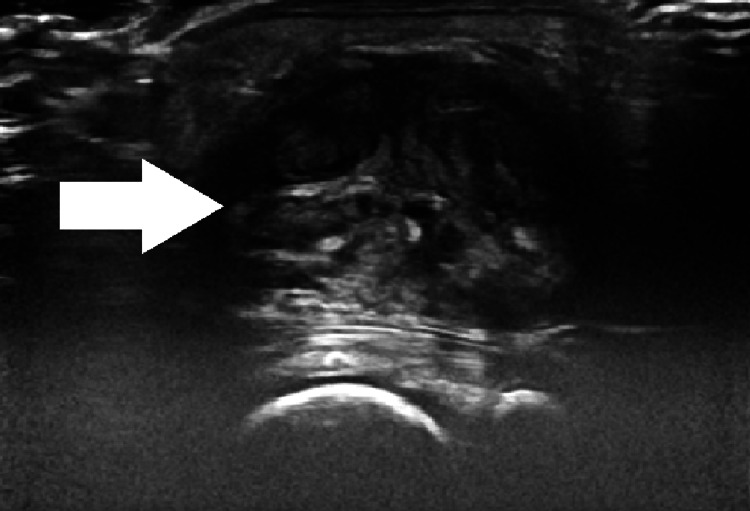
Ultrasound demonstrating a heterogeneous, well-circumscribed swelling in the soft tissue of the right wrist, measuring approximately 41 × 38 mm. The lesion is superficial and localized, with no apparent invasion of adjacent structures.

**Figure 3:**
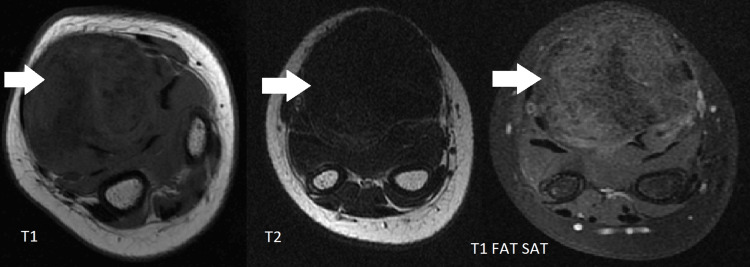
MRI of the wrist in axial T1, T2, and post-contrast T1 fat-saturated sequences demonstrating a roughly ovoid lesion on the anterior aspect of the wrist, with macrolobulated margins. The lesion is isointense on T1 with internal hypointense areas, markedly hypointense on T2, and shows mild, progressive enhancement following contrast administration, suggestive of a fibrous nature.

Ultrasound was performed and showed a heterogeneous, well-circumscribed swelling, measuring 41 × 38 mm, located in the soft tissue of the right wrist anterior surface (Figure 2). The X-ray did not show any associated bone damage. Wrist MRI performed with axial T1- and T2-weighted sequences, as well as post-contrast T1 fat-suppressed imaging, demonstrated a roughly ovoid lesion located on the volar aspect of the wrist, with macrolobulated margins measuring 43 × 41 mm. The lesion appeared isointense on T1 sequences with internal hypointense areas, markedly hypointense on T2, and showed mild, progressive enhancement following contrast administration, suggestive of a fibrous nature (Figure 3). Serum parameters were within normal limits.

**Figure 4:**
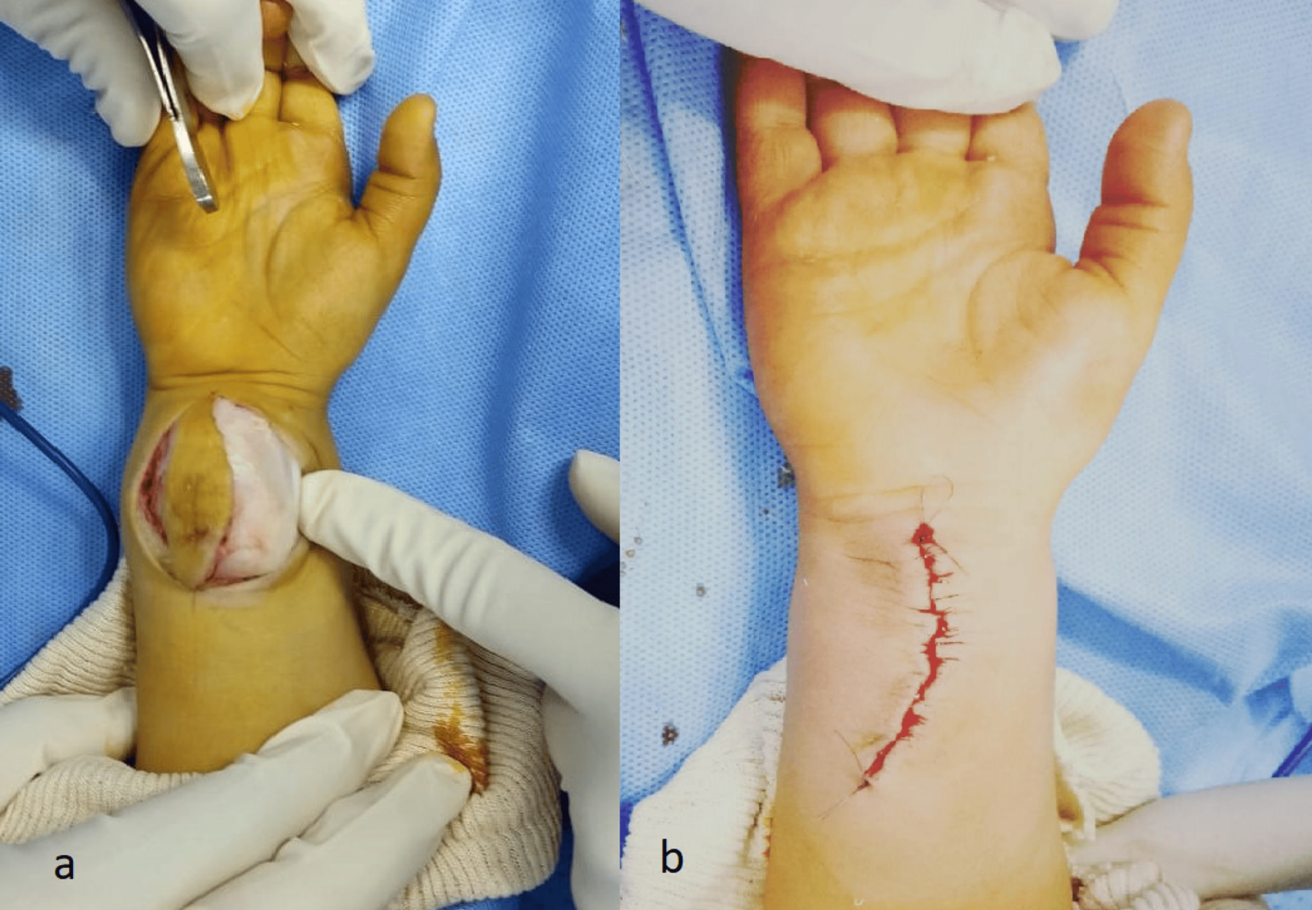
(a) Intraoperative view demonstrating the soft tissue swelling, which is not in contact with bone or tendons. (b) Intraoperative view showing the final appearance of the wrist after complete local excision of the mass.

A surgical biopsy was performed through a direct anterior approach, sampling a fragment measuring 0.8 × 0.5 cm. The histological appearance revealed intersecting bundles of collagen, containing bland fibroblasts and myofibroblasts, devoid of cytologic atypia, along with sparse areas of mature adipose tissue, and finger-like projections of infiltrating fibrous tissue into surrounding fat. A few mesenchymal cells were also seen. Mitosis and necrosis were absent. Immunohistochemistry demonstrated CD34 positivity in the undifferentiated mesenchymal areas, along with smooth muscle actin (SMA) positivity in the spindle cells. These findings excluded differential diagnosis, which included other soft tissue swellings such as lipofibromatosis, lipomatosis, rhabdomyosarcoma, and teratoma. A diagnosis of infantile fibrous hamartoma was thereby confirmed. A marginal excision was performed. The soft tissue swelling was without any contact with bone or tendons (Figure 4).

**Figure 5:**
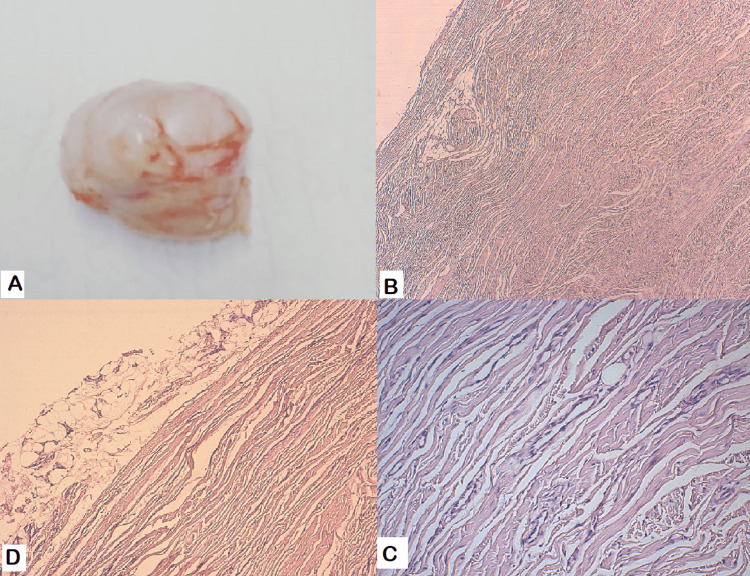
(a) Macroscopic appearance of the resected tumor. (b) Benign tumoral proliferation composed of coarse bundles of collagenous fibrous tissue, containing fibroblasts and myofibroblasts. Area of mature adipose tissue can be seen. (c) High magnification of mesenchymal cells interspersed among bundles of collagenous fibrous tissue. (d) The amount of mature adipose tissue is variable, and can be the predominant component or very sparse.

The immediate postoperative care was uneventful without hemorrhagic or infectious complications. The pathologic examination of the excised mass confirmed the diagnosis of FHI (Figure 5).

The outcome was favorable with recovery of the normal range of motion and pain-free mobility. After 12 months of follow-up, clinical surveillance did not show any signs of recurrence.

## Discussion

Hamartomas are benign tumors arising during fetal life from the abnormal growth of mesenchymal-derived tissues [4]. In 1956, R.D.K. Reye reported a series of six infants under the age of two years with subdermal fibromatous tumors of identical histologic structure [5]. Enzinger in 1965 reported 30 cases with the same clinical and histologic features as the series published by Reye, renaming the condition “fibrous hamartoma of infancy [6].

FHI represents only 0.02% of all benign tumors developing in soft tissues. Usually seen under the age of two years, it can also occasionally present at birth, with a predilection for males, with a ratio of one male to two females. The most common localizations are the axilla, extremities, urogenital area, and abdomen. However, in 50% of cases, this tumor can be located at unusual sites [1,2,7]. In most cases reported in the literature, the clinical presentation comprises a single, painless, mobile, well-limited soft tissue mass, with a diameter not exceeding 5 cm [8]. Few studies have reported cases of FHI with a painful or rapidly growing swelling associated with warmth, tenderness, hypertrichosis, or hyperpigmentation [8-10]. In our case, a one-year-old boy presented with a mass located on the right wrist, measuring 5 × 4 cm. The swelling was solid, mobile, well-limited, and pain-free, which corresponded to the clinical presentation commonly reported in the literature.

On radiographs, as FHI typically appears as a nonspecific soft tissue swelling, it has limited diagnostic value. CT and MRI play an important role in determining the origin and analyzing the surrounding structures by allowing for slices in different planes. FHI appears as a round or oval soft tissue swelling, lobulated, and well-circumscribed with a signal similar to adipose or fibrous tissue [11]. According to adipose and muscle percentage on CT or MRI, FHI are classified as non-balanced or balanced type. In the non-balanced type, one of the two components is predominant and the other one subordinate. However, in the balanced type, the proportion of adipose and muscle is equal [12]. Our case was classified as a non-balanced fibrous hamartoma due to the predominance of the muscle component.

Current surgical recommendations advocate local excision with a 1 cm surgical margin as the treatment of choice, both to rule out malignancy and prevent recurrence. The surgical margin cannot be respected if the excision would be mutilating or if it is necessary to preserve vital structures [3,8]. Tyrosine kinase inhibitors can be used as a therapeutic alternative if total exision is not possible [13]. Enzinger reported a recurrence rate of 16% in a series of 30 cases due to incomplete excision [6]. In a series of 145 cases reported by Al-Ibraheemi, follow-up information was available for only 52 patients, recurrence was noted only in two cases, and the rate of recurrence was about 1% [2]. After 12 months of follow-up, our case did not show any signs of recurrence, thereby emphasizing the importance of complete surgical excision with negative margins whenever feasible.

Definitive diagnosis of FHI is established through histopathological examination and immunohistochemistry [7,14]. Grossly, FHI usualy present as a firm, poorly circumscribed mass with a shiny gray-white appearance. They usually measure 2 to 10 cm, with a median of 5 cm. The histological appearance is characterized by a triphasic histologic pattern composed of bland fibroblastic/myofibroblastic fascicles, mature adipose tissue, and primitive myxoid mesenchymal cell nodules often disposed in a myxoid stroma. Finger-like projections of fibrous tissue infiltrating into surrounding adipose tissue are usually present. Mitosis and necrosis are absent or rare [15,16]. The muscular layer of blood vessels shows positive SMA staining, endothelial cells are CD34-positive, whereas primitive mesenchymal cells are SMA-negative and CD34-positive [7].

The differential diagnoses include fibromatosis, which typically occurs in the fingers and toes. On microscopy, they are characterized by spindle-shaped cells embedded within infiltrative collagenous stroma. Lipofibromatosis is seen in childhood with a predilection for feet and hands, presenting as hyperchoic lesions with variable vascularity without cystic components. They are defined by the absence of immature mesenchymal elements on microscopy. Rhabdomyosarcoma presents as a small round cell with a high ratio of nucleus to cytoplasm, showing positivity for desmin and myoD1. Rarely encountered in this location, teratomas, on the other hand, are known to exhibit a wide range of appearances depending on the germ layer that constitutes the majority of the tumor, but they should be considered as a potential differential diagnosis. Congenital fibrosarcoma has a myxoid appearance and is composed of pale stroma separating small, spindle-shaped cells. The *ETV6-NTRK3* gene fusion allows to confirm this diagnosis [7,17].

## Conclusions

FHI is an uncommon, benign soft tissue tumor, typically presenting during the first two years of life. The clinical characteristics are polymorphic and non-specific; hence, MRI and CT are of interest. Local excision is the most effective treatment. The diagnosis of FHI is only confirmed by the pathological examination of the excised mass and immunohistochemical analysis.
